# Criticality as a signature of healthy neural systems

**DOI:** 10.3389/fnsys.2015.00022

**Published:** 2015-02-25

**Authors:** Paolo Massobrio, Lucilla de Arcangelis, Valentina Pasquale, Henrik J. Jensen, Dietmar Plenz

**Affiliations:** ^1^Department Informatics, Bioengineering, Robotics, System Engineering (DIBRIS), University of GenovaGenova, Italy; ^2^Department Industrial and Information Engineering, Second University of NapoliNapoli, Italy; ^3^Fondazione Istituto Italiano di TecnologiaGenova, Italy; ^4^Department of Mathematics, Centre for Complexity Science, Imperial College of LondonLondon, UK; ^5^Section on Critical Brain Dynamics, National Institute of Mental Health (NIH)Bethesda, MD, USA

**Keywords:** criticality, neuronal avalanches, critical exponents, power law, network dynamics, healthy neural systems

This Research Topic in “Frontiers in Systems Neuroscience” contains a collection of original contributions and review articles on the hypothesis that the normal, healthy brain resides in a critical state. The hypothesis that brain activity, or specifically, neuronal activity in the cortex, might be critical arose from the premise that a critical brain can show the fastest and most flexible adaptation to a rather unpredictable environment (for review see Chialvo, 2010). Over the last decade, numerous signatures of criticality have been identified in brain activity. Some of the most striking examples are the probability distributions of size and duration for intermittent spontaneous activity bursts during ongoing activity in the cortex (Beggs and Plenz, 2003). These distributions have been found to follow power laws, which are conserved across species [rat: (Gireesh and Plenz, 2008); non-human primate: (Petermann et al., 2009; Yu et al., 2011); MEG: (Poil et al., 2012; Palva et al., 2013; Shriki et al., 2013); EEG: (Meisel et al., 2013); fMRI: (Tagliazucchi et al., 2012; Haimovici et al., 2013)] and experimental preparations, spanning from reduced *in vitro* models [i.e., acute and organotypic slices (Beggs and Plenz, 2003) and dissociated cultures (Pasquale et al., 2008)] to *in vivo* animal models (Gireesh and Plenz, 2008; Petermann et al., 2009; Ribeiro et al., 2010). These scale-free activation patterns, called *neuronal avalanches*, provide evidence for criticality in the brain.

The bold claim that the brain is critical has elicited a healthy dose of skepticism and critique, often on technical grounds. For example, power laws are ubiquitous in nature, can potentially emerge from noise, and might not be particular to brain function (Touboul and Destexhe, 2010). This critique has refocused the debate on the specific, shallow exponents found for avalanche power laws, which demonstrate that unique, long-range spatial correlations are introduced by these dynamics, which require precisely balanced, weak interactions that differ from noise (Klaus et al., 2011). Similarly, discussion about the proper power law model and functional fit (Clauset et al., 2009; Dehghani et al., 2012) has highlighted the importance of careful identification of power law cut-offs in avalanche distributions, and their correct incorporation into appropriate statistical models (e.g., Langlois et al., 2014; Yu et al., 2014). Importantly, alternative approaches to avalanche dynamics using temporal scaling (Hardstone et al., 2012) and spatial scaling of fluctuations in ongoing human brain activity (Haimovici et al., 2013) have brought further support to the hypothesis of criticality in the brain.

As evidenced in the contributions to this Special Topic issue, the exploration and examination of brain activity in the framework of criticality represents a highly active, ongoing field of research. It has been shown that the distribution of silent times between consecutive avalanches displays a non-monotonic behavior (due to the slow alternation between up- and down-states, Scarpetta and De Candia, 2014), with a power law decay at short time scales (Lombardi et al., 2014). Further analyses (Lombardi et al., 2012) demonstrate that avalanche size and inter-avalanche silent times are correlated, and highlight that avalanche occurrence exhibits the characteristic periodicity of θ and β/*γ* oscillations. This observation is in line with pharmacological results that connect nested oscillations and neuronal avalanches in cortex (Gireesh and Plenz, 2008). Experimental observations of long-term temporal correlations (Botcharova et al., 2014) in fluctuations of phase synchronization in EEG and MEG signals suggest that the driving mechanisms behind avalanche activity are non-local, with all scales contributing to system behavior. Indeed, an important “keyword” that characterizes such scale-free systems is the presence of a critical point, indicating the existence of a critical branching process as underlying structure that sustains this kind of dynamics. As it emerges in Yu et al. (2013), the ongoing resting activity in cortical networks organizes close to an effective thermodynamic critical point, suggesting the possibility that a critical state may in effect be described by methodology from thermodynamic equilibrium. As reviewed in Hesse and Gross (2014), a critical system displays optimal computational properties, indicating that criticality has been evolutionarily selected as a useful feature for the nervous system. The progress in the field of criticality and brain dynamics is further demonstrated by the fact that current discussions, rather than rejecting criticality altogether, are often focused on the proximity of brain dynamics to the critical point under different conditions. Based on *in vivo* recordings of extracellular spiking activity and modeling work, it has been concluded that the brain does not reflect a critical state, but its emergent dynamics might self-organize to a slightly sub-critical regime (Priesemann et al., 2014). To reside in such a regime can be considered an advantage, since it might prevent brain activity from becoming epileptic, which has been associated with supercritical dynamics (Meisel et al., 2012). Based on modeling (Tomen et al., 2014), it has been suggested that cortical networks, by operating at the sub-critical to critical transition region, could dramatically enhance stimulus representation.

Thus, if the brain works close to or at a critical point, it is interesting to investigate the role of criticality on cognition and long-term temporal correlations observed in behavioral studies (Papo, 2014). Moreover, little is known about the causes and/or consequences of a loss of criticality, and its relation with brain diseases (e.g., epilepsy). The study of how pathogenic mechanisms are related to the critical/non-critical behavior of neuronal networks would likely provide new insights into the cellular and synaptic determinants supporting the emergence of critical-like dynamics and structures in neural systems. At the same time, the relationship between disrupted criticality and impaired behavior would help clarify the role of critical dynamics in normal brain functioning. In this Research Topic, (Tinker and Perez Velazquez, 2014) studied whether power law scaling can be achieved in the distribution of phase synchronization derived from MEG recordings, acquired from children with or without autism performing executive function tasks. Interestingly, (Roberts et al., 2014) point out an issue not well explored in previous works: i.e., that existing models lack precise physiological descriptions for how the brain maintains its tuning near a critical point. The authors claim that a missing fundamental ingredient is a formulation of the reciprocal coupling between neural activity and metabolic resources. Recent findings are aligned with the author's idea, which emerged from the analysis of disorders involving severe metabolic disturbances and altered scale-free properties of brain dynamics.

The hypothesis that cortical dynamics resides at a critical point, at which information processing is optimized, has refocused attempts to explain the tremendous variability in neuronal activity patterns observed in the brain at all scales. Over the last several years, this hypothesis has given rise to numerous conferences and workshops on the brain and criticality (Plenz and Niebur, 2014). The current Research Topic continues the endeavor to explore one of the most exciting current concepts on brain function.

## Conflict of interest statement

The authors declare that the research was conducted in the absence of any commercial or financial relationships that could be construed as a potential conflict of interest.
